# The role of carbon ion radiotherapy for unresectable locally recurrent rectal cancer: a single institutional experience

**DOI:** 10.1186/s13014-020-01653-w

**Published:** 2020-08-28

**Authors:** Xin Cai, Yueyao Du, Zheng Wang, Ping Li, Zhan Yu, Qing Zhang, Zhen Zhang

**Affiliations:** 1 Department of Radiation Oncology, Shanghai Proton and Heavy Ion Center, 4365 Kang Xin Road, Shanghai, 201321 China; 2 Shanghai Engineering Research Center of Proton and Heavy Ion Radiation Therapy, 4365 Kang Xin Road, Shanghai, 201321 China; 3 Department of Breast Surgery, Renji Hospital, School of Medicine, Shanghai Jiao Tong University, Shanghai, 200127 China; 4 Shanghai Proton and Heavy Ion Center, Fudan University Cancer Hospital, 4365 Kang Xin Road, Shanghai, 201321 China

**Keywords:** Carbon-ion, Rectal cancer, Pelvic radiation, Local recurrence, Particle therapy

## Abstract

**Background:**

Treatment for locally recurrent rectal cancer after surgery is still a challenge. With the physical and biological advantages, carbon-ion radiotherapy (CIRT) could be a choice for these patients. The purpose of this study was to investigate the efficacy and safety of CIRT for unresectable locally recurrent rectal cancer in Chinese patients.

**Methods:**

Date from 25 patients with unresectable locally recurrent rectal cancer treated by CIRT from July 2015 to April 2019 were analyzed retrospectively. The endpoints of this study were overall survival (OS), local control (LC) and acute and late toxicity.

**Results:**

With the median follow-up of 19.6 (range 5.1–52.5) months, data of all 25 patients were collected. Median prescribed dose for tumor was 72Gy (relative biologic efficacy (RBE)) (range 48–75.6Gy (RBE)). The LC rates at 1 and 2 years were 90.4 and 71.8%. Overall LC at 1- and 2-year were 76.2 and 30.5% for 9 patients whose prescribed tumor doses of CIRT< 66 Gy (RBE), 100 and 100% for 16 patients whose prescribed doses of CIRT≥66 Gy (RBE). Patients received ≥66 Gy (RBE) had obviously better LC rates than those received < 66 Gy (RBE) (*P* = 0.001). The OS rates at 1 and 2 years were 82.9 and 65.1%, respectively. No acute toxicity over grade 2 was observed, grade 3 late toxicity were observed in 3 patients: gastrointestinal toxicity (*n* = 1), neuropathy (n = 1), pelvic infection (n = 1). No Grade 4 or higher toxicity was observed.

**Conclusion:**

Our study shows that CIRT is effective for unresectable locally recurrent rectal cancer patients with acceptable toxicity.

## Introduction

Local recurrence (LR) presents a challenge after combined modality treatment for rectal cancer. Although multiple strategies including total mesorectal excision (TME), radiation therapy as well as chemotherapy has been shown to improve clinical outcomes, it remains a significant problem in practice [1, 2], and about 4–15% of the patients with rectal cancer will suffer from LR [3–7].

Several studies have shown that LRs after definitive surgery occur in the central or posterior pelvis, with the presacral-perineal space being identified as the primary site of recurrence. Salvage surgical therapy remains the mainstay for these LR lesions, offering the best chance for cure. However, only 20–30% of patients receive a potentially curative operation due to its high rates of complication and operative mortality [8, 9]. Radiotherapy may be one of the alternative treatment strategies that can be employed to the ones that surgical resection is unfeasible. As we know, a higher radiation dose to the area may increase local control. But LR disease treated by conventional external beam radiation (EBRT) is often limited by surrounding dose-limiting structures such as small bowel and bladder. Furthermore, LR disease may present with a large fraction of hypoxic cells, which are always resistant to conventional irradiation [10].

Compared with conventional photon therapies, particle radiotherapy, in particular carbon-ion radiotherapy (CIRT), has its unique physical and biologic advantages in the treatment of local recurrent rectal cancer [11]. From physical aspects, CIRT is characterized by improved dose distribution and minimization of dose to surrounding normal tissues. Numerous studies [12–14] has shown that CIRT is effective in treating various solid carcinomas. As a promising evolving modality in radiotherapy, CIRT has special biological advantage to overcome the radioresistance of hypoxic cells [15]. Previous clinical data further concluded that oxygen status was not the independent predictor of local control with the use of CIRT [16], which indicated that CIRT might be an effective way to increase radiosensitivity for local recurrent disease. The objective of this trial was to assess the effectiveness and safety of CIRT in the treatment of recurrent rectal cancer in Chinese patients.

## Materials and methods

### Patient eligibility

From July 2015 until March 2019, 25 unresectable pelvic local recurrent rectal cancer patients were treated by CIRT. Initially, all the patients had pathologically confirmed rectal adenocarcinoma (including mucious adenocarcinoma and signet-ring cell carcinoma) and underwent a curative resection of their primary disease and regional lymph nodes, without gross or microscopic residual disease. Clinical recurrent diagnosis should be confirmed by biopsy or meet at least two of the three criteria: 1) relative tumor markers elevated; 2) hypermetabolic lesion in positron emission tomography (PET) imaging; 3) imaging follow-up revealed gradually enlargement of occupying lesion. Most of the patients (22 of 25 patients) had isolated pelvic or lymph node recurrence without distant metastasis verified by computed tomography (CT) or magnetic resonance imaging (MRI) or PET imaging. Among the 25 patients, 3 had received chemotherapy due to unresectable lesions of lung metastasis and evaluated as stable disease (SD) at least three months before the treatment of CIRT. Surgical resection of LR was evaluated as unfeasible by experienced surgeons. Patients were required to have Eastern Cooperative Oncology Group performance status ≤2, life expectancy ≥12 months, and pretreatment evaluation consisted of a complete history and physical examination, complete blood count (CBC), renal and liver function tests, chest CT, ultrasound of abdomen, and MRI/CT of the pelvis or whole body PET-CT.

The excluded criteria included: 1) a previous history of other malignancy; 2) receiving more than one prior radiotherapy in the same site or time to last radiotherapy is less than 1 year; 3) acute bacterial or fungal infection. If the lesion of the local recurrence was too close (less than 5 mm) to vital organs (bladder, digestive tract) were also excluded.

This study was approved by the ethics committee. Before starting CIRT, patients were informed of their disease status, the risks and benefits and the expense of CIRT. Written informed consent was obtained from each subject.

### Carbon-ion radiotherapy

All 25 patients received carbon-ion external irradiation by Siemens particle therapy device. Carbon-ion irradiation was performed daily, five days a week, with a total of 16 to 21 fractions in 22 to 30 days. The gross tumor volume (GTV) was defined as the area of contrast enhancement on T1-weighted MR-imaging. The clinical target volume (CTV) was defined as the GTV adding a safety margin of 5-10 mm adapted for organ at risk (OARs). The planning target volume (PTV) was defined as 5 mm margin added to the CTV.

The median GTV dose of CIRT was 72Gy (RBE)(48–75.6Gy (RBE))and prescribed to the 95% isodose line. For 8 patients without prior radiotherapy, the prescribed doses for GTV were 57-72Gy (RBE), 19–20 fractions at 3, 3.3 or 3.6 Gy (RBE) per daily fraction. For other 17 patients with prior radiotherapy over 1 year before CIRT, dose given for CTV were 48–75.6Gy (RBE), 16–21 fractions at 3, 3.3 or 3.6 Gy (RBE) per daily fraction. For 18 patients (13 with prior radiotherapy) treated with SIB, the median dose for GTV in these 18 patients was 72Gy (RBE) (range:57.6 to 75.6Gy (RBE)), at 3.3 or 3.6Gy (RBE) per daily fraction in 16–21 fractions.

### Chemotherapy

Concurrent chemotherapy was applied in 7 patients whose total GTV dose was less than 60Gy (RBE) (capecitabine: 825 mg/m^2^ twice daily, 5 days per week) and CIRT. Considering of the gastrointestinal toxicity caused by chemotherapy and CIRT, concurrent chemotherapy was not given to patients who received higher dose. Systemic treatment was recommended for patients as adjuvant treatment.

### Treatment results and evaluation of adverse events

#### Follow-up

All patients were followed up according to the protocol. The first follow-up is one month after CIRT and then patients were followed up every 3 months in the initial 2 years and every 3–6 months thereafter. Post treatment evaluation included pelvic MRI or CT or PET scans, et al. Changes in the tumor diameter before and after treatment were evaluated in accordance with the Response Evaluation Criteria in Solid Tumors (RECIST) scoring system (version 1.0). Complete response (CR) was defined as disappearance of the target lesions. Partial response (PR) indicates at least a 30% decrease in the sum of longest diameter (LD) of the target lesion, taking as reference the baseline sum LD. SD ranged from a 30% decrease to a 20% increase in size. Progressive disease (PD) was described as at least a 20% increase in the sum of the LD of the target lesion.

#### Adverse effects

Acute toxicity from treatment was classified according to the National Cancer Institute Common Toxicity (CTCAE) Version 4.03. Acute toxicity was defined as symptoms first occurring or lasting < 90 days after the completion of radiotherapy.

#### Statistics

Time to locoregional failure and distant metastases was measured from the completion of EBRT until documented treatment failure. Local control (LC) was defined as the absence of notable local disease recurrence based on MRI/CT, and/or PET scans. Local recurrence indicated measurable lesions occurring in the irradiated tumor bed. The LC rate and survival rate curves were estimated using the Kaplan-Meier method. All statistical analyses were calculated by Stata 16.0 (StataCorp LP, College Station, TX, USA).

## Results

### Clinical characteristics of patients

From July 2015 to April 2019, 25 patients with 25 lesions were enrolled in this study. Detailed characteristics of the patients enrolled in this study are shown in Table 1. Median patient age was 53 years (range 32 to 72). Relapse locations included the presacral region (*n* = 11), pelvic sidewalls (*n* = 9), perineum (*n* = 4), and colorectal perianastomosis (n = 1).

**Table 1:** Patient characteristics

Characteristics	No. of patients (%)
**Age, years**	
Median	53
Range	32–72
**KPS**	
80	7 (28.0%)
90	14 (56.0%)
100	4 (16.0%)
**Gender**	
Male	19 (76.0%)
Female	6 (24.0%)
**Primary tumor operation**	
abdominoperineal excision	13 (52.0%)
low anterior resection	10 (40.0%)
Hartmann’s resection	2 (8.0%)
**Prior pelvic radiation therapy**	
Yes	17 (68.0%)
No	8 (32.0%)
**Dose of prior pelvic radiation therapy**	
Median	50Gy
Range	27-60Gy
**Tumor sites**	
presacral	11 (44.0%)
side wall	9 (36.0%)
perineal	4 (16.0%)
perianastomosis	1 (4.0%)
**Tumor size**	
range	6.1–334.1 (ml)
average	84.5 (ml)
**Total Dose of Carbon Ion**	
< 66Gy (RBE)	9 (36.0%)
≥ 66Gy (RBE)	16 (64.0%)

The median follow-up duration for all the patients was 19.6 months (range, 5.1–52.5 months). All patients completed the entire treatment course.

After CIRT, 3 patients with unresectable distant metastasis diagnosed before CIRT continued chemotherapy. Among the rest of 22 patients, 18 patients received at least 4 cycles of fluorouracil-based chemotherapy. 4 patients did not receive chemotherapy, among them 2 were considered not tolerant to chemotherapy. 1 patient with small tumor size was suggested to follow up only. 1 patient refused to receive chemotherapy.

### Tumor response

Treatment response was evaluated in all 25 patients (Table 2), four of 25 (16%) had PR and twenty-one of 25 (84%) patients had SD as soon as the treatment of CIRT was finished. Until the last follow-up in the end of March 2020, changes in the tumor were evaluated by image again and the results showed that 2 (8%) and 7 (28%) patients were evaluated as CR and PR, 10(40%) patients were considered as SD, and 6(24%) patients were considered as PD for their pelvic lesions. One of the patients defined as PR by image received surgery due to gastrointestinal toxicity, and the pathological report showed there was no tumor left and he achieved pathological complete response (pCR). Of the 16 patients with symptomatic response before CIRT, 15 (93.8%) had improvement in their pain.

**Table 2:** Summary of clinical findings

Parameter	No. of patients (%)
**Response (*n*** **= 25)**	
Pathologic complete response	1 (4.0%)
Clinical complete response	2 (8.0%)
Partial response	6 (24.0%)
Stable disease	10 (40.0%)
Progressive disease	6 (24.0%)
**Symptom relief (*n*** **= 16)**	
Complete relief	9 (56.3%)
Partial relief	6 (37.5%)
No relief	1 (6.2%)

There were 3 patients who had distant metastases before the treatment of CIRT. During the follow-up, 9 of the other 22 patients developed distant metastases, among which 4 patients experience lung metastases, 3 patients experience bone metastases, 1 patient experience retroperitoneal lymph node metastasis and 1 patient experience both lung and bone metastases.

**Fig. 1:**
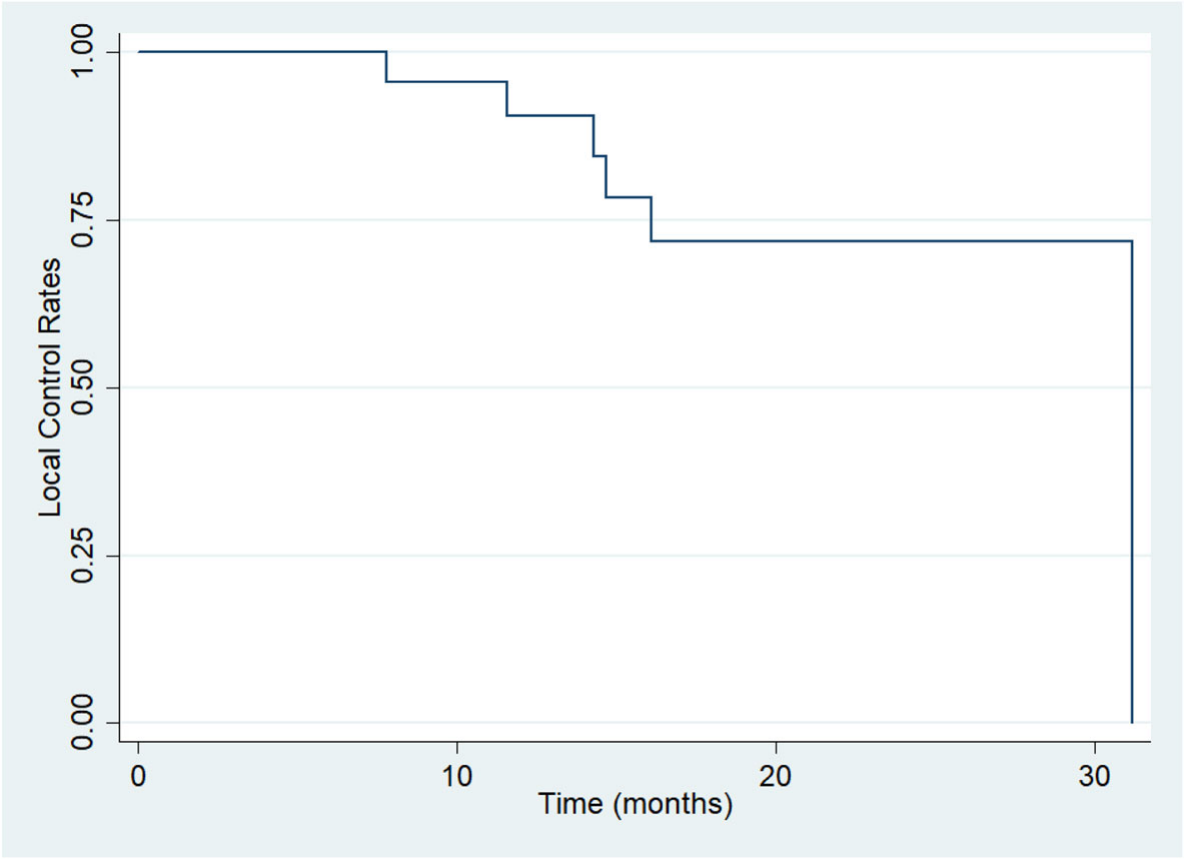
Kaplan-Meier estimates of local control rate for the 25 patients with recurrent rectal cancer after the treatment of CIRT

**Fig. 2:**
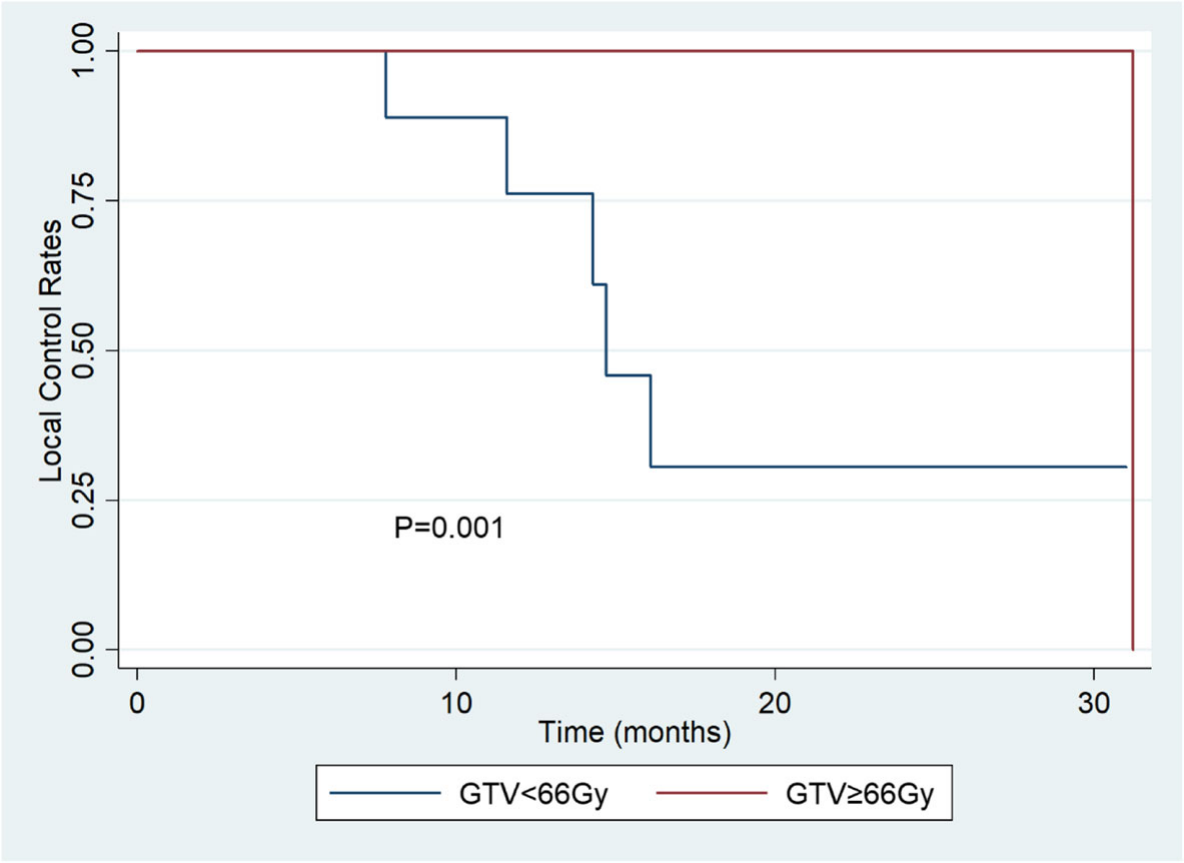
Kaplan-Meier estimates of local control rate for patients whose prescribed tumor doses of CIRT< 66 Gy (RBE) and ≥ 66 Gy (RBE)

The 1- and 2-year LC rates were 90.4% (95% CI, 66.8 to 97.5%) and 71.8% (95% CI, 44.4 to 87.4%) (Fig. 1). Overall LC at 1- and 2-year using the Kaplan Meier method were 76.2% (95% CI, 33.2 to 93.5%) and 30.5% (95% CI, 4.5 to 63.4%) for patients whose prescribed tumor doses of CIRT< 66 Gy (RBE), 100 and 100% for patients whose prescribed doses of CIRT≥66 Gy (RBE). Patients received ≥66 Gy (RBE) had obviously better LC rates than those received < 66 Gy (RBE) (*P* = 0.001) (Fig. 2). The 1- and 2-year LC rates were 86.2% (95% CI, 55.0 to 96.4%) for 17 patients who received re-irradiation for pelvic recurrence of rectal cancer.

**Fig. 3:**
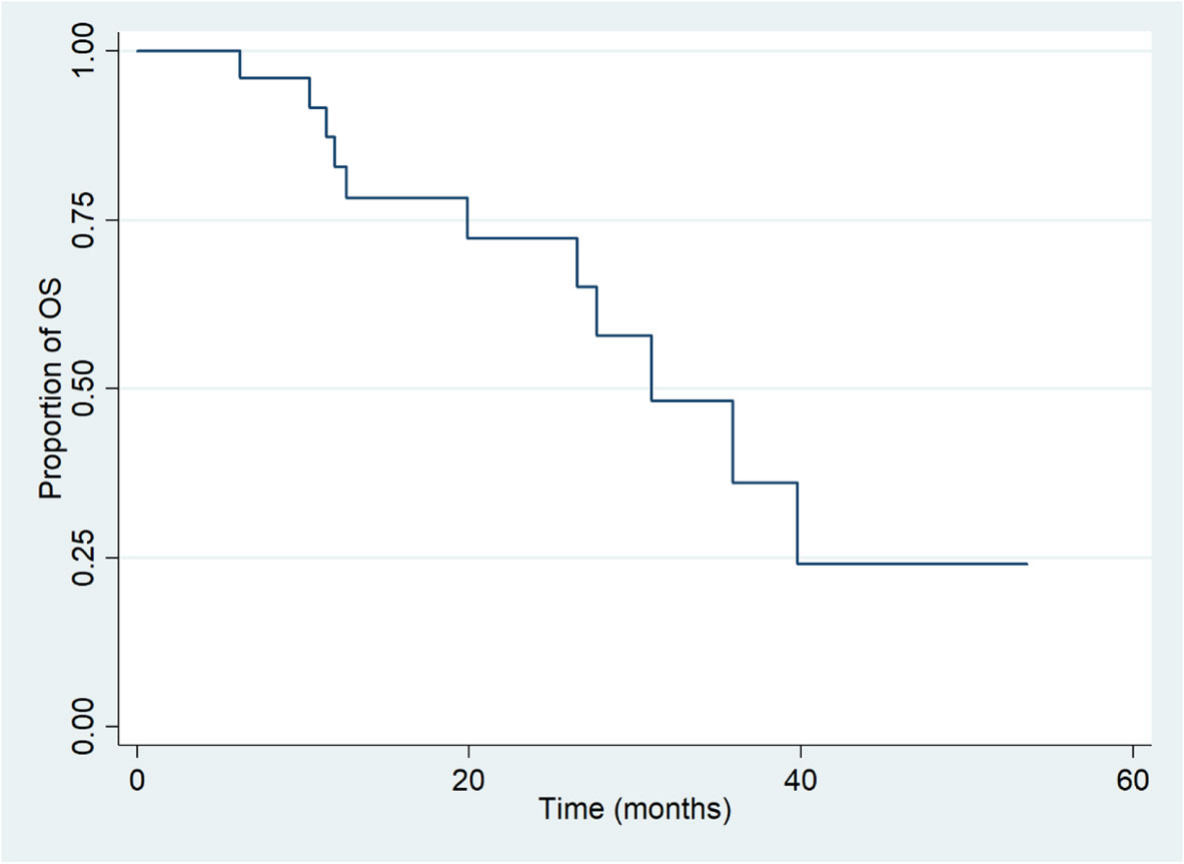
Kaplan-Meier estimates of overall survival rate for the 25 patients with recurrent rectal cancer after the treatment of CIRT

The 1- and 2-year overall survival rate were 82.9% (95% CI, 60.6 to 93.2%) and 65.1% (95% CI, 39.0 to 82.2%) (Fig. 3). Overall survival rate at 1- and 2-year using the Kaplan Meier method were 77.8% (95% CI, 36.5 to 93.9%) and 55.6% (95% CI, 20.4 to 80.5%) for patients whose prescribed doses of CIRT< 66 Gy (RBE), 86.5% (95% CI, 55.8 to 96.5%) and 69.2% (95% CI, 25.5 to 90.6%) for patients whose prescribed doses of CIRT≥66 Gy (RBE) (Table 3).

**Table 3:** Local control rate and overall survival rate of the patients receiving CIRT

	1 year	2 year
**LC**	90.4%	71.8%
< 66 (RBE)	76.2%	30.5%
≥ 66 (RBE)	100%	100%
**OS**	82.9%	65.1%
< 66 (RBE)	77.8%	55.6%
≥ 66 (RBE)	86.5	69.2%

### Toxicity

Acute and late toxicity of the 25 cases receiving CIRT are described in Table 4. No grade > 3 acute toxicities were observed. Grade 3 late toxicity were observed in 3 patients: gastrointestinal toxicity (*n* = 1), neuropathy (*n* = 1), pelvic infection (*n* = 1).

**Table 4:** Acute and late toxicities of the patients receiving CIRT

	Acute (NCI-CTC)						Late (RTOG/EORTC)					
Toxicity	n	Gr0	Gr1	Gr2	Gr3	Gr4	n	Gr0	Gr1	Gr2	Gr3	Gr4
Skin	25	22	1	2	0	0	25	22	3	0	0	0
GI tract	25	24	1	0	0	0	25	24	0	0	1	0
Neuropathy	25	25	0	0	0	0	25	24	0	0	1	0
Pelvic infection	25	25	0	0	0	0	25	24	0	0	1	0
Hematological	25	9	11	5	0	0	25	25	0	0	0	0
Others	25	24	1	0	0	0	25	25	0	0	0	0

## Discussion

The present study firstly reported the clinical outcomes of 25 patients treated with CIRT for pelvic recurrence of rectal cancer in Chinese patients. With a median follow-up of 19.6 months, the 2-year LC rate and OS rate were 71.8 and 65.1%, respectively. CIRT achieved favorable LC and survival comparable to surgery. Otherwise, adverse events were self-limited during the treatment of CIRT, without any grade 4 or higher toxicity. The results are promising and suggest that CIRT is an optional therapy for patients with pelvic recurrence of rectal cancer.

Previous clinical results for locally recurrent rectal cancer (LRRC) treated by CIRT were reported by Heidelberg Ion Beam Therapy Center (HIT) in Germany and National Institutional of Radiological Sciences Hospital (NIRS) in Japan. In HIT’s study, 19 patients received carbon ion irradiation to treat LRRC. All patients had a history of surgery and pelvic radiotherapy of at least 50.4Gy. The range of CIRT dose was 36-51Gy (RBE), 3Gy (RBE) per fraction. With median follow-up of 8 months, local progression-free survival was 20.6 months [14]. In NIRS’s study [11], Shinoto et al. investigated the efficacy and safety of CIRT for LRRC. 224 patients were enrolled in the study and the prescribed dose was 70.4 or 73.6Gy (RBE) in 16 fractions. The results showed that LC rates were 93% at 3 years and 88% at 5 years. OS rates were 73% at 3 years and 51% at 5 years. The prescribed dose of CIRT was relatively low and the follow-up duration was short in HIT’s study. Compared with the results of the previous two studies, as the first study in patients receiving CIRT for LRRC in China, our study yielded acceptable and encouraging results in the efficacy of CIRT in treating LRRC.

For LRRC, there are other radiation modalities except CIRT. Cai et al. evaluated the efficacy and safety of irinotecan and capecitabine with concurrent intensity-modulated radiation therapy (IMRT) for the treatment of recurrent rectal cancer without prior pelvic irradiation. Radiotherapy was delivered to the pelvis, and IMRT of 45 Gy (1.8 Gy per fraction), followed by a boost of 10 Gy to 16 Gy, was delivered to the recurrent sites. For medically fit patients without extra-pelvic metastases, they would be recommended to receive radical surgery. After chemoradiation, the LC rates at 1 and 3 years were 74.2 and 33.9%, the OS rates at 1 and 3 years were 80.1 and 36.5%, respectively [17]. 2 patients experienced grade 4 leukopenia. No acute toxicity over grade 3 was reported. Sun et al. evaluated the efficacy and treatment-related toxicity of accelerated hyperfractionation field-involved re-irradiation combined with concurrent capecitabine chemotherapy for LRRC. Surgery would be performed after radiation if the disease was resectable. 3-year LC and OS were 31.19 and 45.12% [18]. Incidence of grade 3–4 diarrhea and granulocytopenia was 9.7 and 8.3%. Small bowel obstruction was severely late toxicity, and the incidence was 1.4%. Although the LC and OS rates in these two studies were acceptable, it should be noted that after IMRT and hyperfractionation re-irradiation, radical surgery would be performed if the lesion was evaluated as resectable. Defoe et al. evaluated the safety and efficacy of stereotactic body radiotherapy (SBRT) in LRRC patients with prior pelvic radiation. 11 patients were treated with 36 Gy in 3 fractions and 3 patients were treated with single fraction of 12, 16 or 18 Gy. 1y- and 2y- LC rates were 90.9 and 68.2% and the 1y- and 2y- OS rates were 90 and 78.8%, respectively. No acute toxicity over grade 3 was observed [19]. Compared with conventional photon modalities, our results showed that CIRT was effective as a radical treatment modality for LRRC with or without prior pelvic radiation.

In our study, there was a significant correlation between dose and local control rates. Overall LC at 1- and 2-year using the Kaplan Meier method were 76.2 and 30.5% for patients whose prescribed tumor doses of CIRT< 66 Gy (RBE), 100 and 100% for patients whose prescribed doses of CIRT≥66 Gy (RBE). Actually, among patients whose prescribed doses of CIRT≥66 Gy (RBE), only one patient progressed after 31.2 months’ follow-up. In HIT’s study [14], the results showed that applied dose (36Gy (RBE) vs. ≥36Gy (RBE), 20.2 vs. 15.2 months, respectively) were not predictive of local failure. In NIRS’s study [11], no correlation between prescribed dose and local control was noted. It should be noted that in HIT’s study, the applied does was relatively low and in NIRS’s study, the prescribed dose were almost the same. Another concern was the accuracy of CIRT dose deliver to the primary area, while there were RBE variation along the full range of spread-out Bragg peak (SOBP) fields. We confirmed that our planning system already calculated the physics dose based on the different RBE weighting, which indicated that LC might not correlated with the LET-RBE ratio. It was reasonable that in our results, patients received ≥66 Gy (RBE) had obviously better LC rates than those received < 66 Gy (RBE). It suggests that the currently applied dose of CIRT (≥66 Gy) in our center is feasible for patients with LRRC.

The toxicities of CIRT in treating LRRC were mild in our study. Systemic treatment before and after CIRT was suggested for LRRC patients. With less hematological toxicities caused by CIRT, more systemic treatment could be applied to decrease distant metastases, which might have benefits for LRRC patients in OS. Tumors that were close to gastrointestinal tract might cause gastrointestinal toxicity and pelvic infection in our study. Neuroinjury was reported mostly in high biological equivalent dose (BED) radiation therapy such as SBRT and CIRT in Japan. One of the patients enrolled in our study suffered neuropathy 11 months after CIRT. Since our treatment planning system already concerned the RBE variation along the different SOBP location, and the physics dose was determined based on different RBE weighting, it was reasonable not to concern more dose variation to the target volume. By reviewing CT scan, we found that the injured nerve defined by electromyography was located at the high BED dose area. As high LET radiation, carbon ion would present with higher biological effects than Low LET irradiation, such as conventional radiotherapy. The fraction size for this patient was xx, which would translate to a higher biological effective dose delivered to the target volume. Based on LQ model to predict the biologic effectiveness, the biologic equivalent dose of CIRT for this patient corresponded to approximately xx Gy of EBRT delivered in standard 2Gy fractionation. Therefore, we considered that the neuropathy might be due to the higher BED dose and we should pay more attention to the fractionated size of CIRT for organs at risk in the future plans.

As to the selection of patients with locally recurrent rectal cancer, surgery remains the major treatment for resectable diseases. All the participants enrolled in the present study were patients with unresectable locally recurrent rectal cancer and most of them received pelvic radiotherapy before. Considering the risk of re-irradiation, the lesion of the local recurrence should be at least 10 mm to vital organs (bladder, digestive tract). The results of the present study showed that patients received higher prescribed tumor doses of CIRT had obviously better LC rates. In the present study, the prescribed tumor dose of the first patient who achieved CR was 66 Gy (RBE), and it was the reason why we chose 66 Gy (RBE) as a cut-off point. Optimal cut-off point needs to be further explored in the future study.

There are some limitations in the present study, which need to be acknowledged. First, this is a retrospective study with a relatively short follow-up time. Longer follow-up on clinical outcomes is needed. Second, the sample size of this study is small. Larger and well-designed prospective studies are required to further evaluate the role of CIRT in treating LRRC. Third, systemic treatment before or after CIRT might affect OS of LRRC patients, which was not further analyzed in this study.

To sum up, the results of the present study suggest that CIRT is effective for LRRC and can provide LC and OS rates that are comparable to those of the prior studies of CIRT. The incidence of acute and late toxicities was also tolerable. CIRT should be considered as a safe, effective treatment option for LRRC and can provide an alternative to surgery.

## Data Availability

The data and materials of this study are available from the corresponding author on reasonable request.

## Abbreviations

CIRT: carbon-ion radiotherapy
OS: overall survival
LC: local control
RBE: relative biologic efficacy
LR: local recurrence
TME: total mesorectal excision
EBRT: external beam radiaton
PET: positron emission tomography
CT: computed tomography
MRI: magnetic resonance imaging
SD: stable disease
CBC: complete blood count
GTV: gross tumor volume
CTV: clinical target volume
PTV: planning target volume
RECIST: Evaluation Criteria in Solid Tumors
CR: complete response
PR: partial response
LD: longest diameter
PD: progressive disease
CTCAE: National Cancer Institute Common Toxicity
pCR: pathological complete response
LRRC: locally recurrent rectal cancer
HIT: Heidelberg Ion Beam Therapy Center
NIRS: National Institutional of Radiological Sciences Hospital
IMRT: intensity-modulated radiation therapy
SBRT: stereotactic body radiotherapy
BED: biological equralent dose
